# Personalized medicine in Type 2 Diabetes

**DOI:** 10.7603/s40681-014-0008-z

**Published:** 2014-08-06

**Authors:** Wen-Ling Liao, Fuu-Jen Tsai

**Affiliations:** 1 Center for Personalized Medicine, China Medical University Hospital, Taichung, Taiwan; 2 Graduate Institute of Integrated Medicine, China Medical University, Taichung, Taiwan; 3 Department of Medical Research and Medical Genetics, China Medical University Hospital Taichung, Taichung, Taiwan; 4 School of Chinese Medicine, China Medical University, Taichung, Taiwan; 5 Department of Pediatrics, China Medical University Hospital, Taichung, Taiwan; 6 Department of Health and Nutrition Biotechnology, Asia University, Taichung, Taiwan; 7 Department of Medical Genetics, Pediatrics and Medical Research, China Medical University Hospital, No.2 Yuh-Der Road, 404 Taichung, Taiwan

**Keywords:** Genetic, Pharmacogenetics, Diabetes

## Abstract

Type 2 diabetes (T2D) is a global public health concern, its prevalence in Asia, especially Taiwan, rising every year. The risk of developing T2D and diabetes complications is not only controlled by environmental but also by genetic factors. Genetic association studies have shown polymorphisms at specific loci may help identify individuals at greatest risk and response to oral antidiabetic drugs. This review probes effect of genetic profiling on T2D and its complications, using our study population as examples. Also, pharmacogenetics and pharmacogenomics of oral anitdiabetic drug will be explored.

## 1. Introduction

Type 2 diabetes (T2D) is one of the leading health problems worldwide, with the fastest growing incidence of chronic disease in the 21^st^ century. Global diabetic population will increase to 300 million by 2025. T2D is a complex metabolic disorder with high morbidity and mortality [1]. Overall risk of death among people with diabetes is about twice that of non-diabetics in the same age bracket. Most diabetics have one or more micro- or macro- complications as overt or subclinical manifestations during the course of their disease. Large-scale study [2] among over 7,000 patients with T2D in eight European countries concluded that approximately 72% of the participants had at least one complication, 24% both micro- and macrovascular complications. These pose significant public health problems: e.g., a large proportion of blindness, renal replacement therapy, cardiovascular intervention. Owing to increasing prevalence of diabetes, T2D and its complications will wreak substantial negative impact on both overall healthcare expenditures and patients’ quality of life.

T2D is a group of metabolic diseases by chronic hyperglycemia with disturbances of fat, carbohydrate, and protein metabolism resulting from defects in insulin secretion or activity [3]. This disease is heterogeneous in both pathogenesis and in clinical symptoms, a manifestation of multiple interconnected aberrant pathways and numerous molecular abnormalities acting in concert with negative and positive environmental factors. In current medical care, such patients often are treated similarly, with little consideration of individual characteristics that might affect clinical outcome and therapeutic response, despite considerable variation between cases. Prior studies demonstrated heredity playing a key role in both pathogenesis and complications [4,5]. Personalized medicine is a relatively new paradigm of evidence-based medicine, based on the established principle of each individual born with unique biological and genetic characteristics. The risk, progression and treatment of disease would differ based on individual variation; hence determining optimal therapeutic strategy for individual patients is critical.

Previous studies have demonstrated heredity playing a key role in diabetic pathogenesis and complications. Specifically, in the Framingham Offspring Study, subjects of one T2D parent have 3.5-fold risk of diabetes, 6.1-fold compared with the general population if both parents have T2D [4]. Hundreds of genes have cited for susceptibility to T2D and diabetic complications by linkage studies, candidate gene association studies, genome-wide association studies (GWAS) and meta analyses in diverse ethnic groups. We focus on genetic profiling as it affects T2D and diabetic complications. There is considerable variation between patients with the same disease. Some diabetics show no response to treatment, others rapidly respond. Underlying this variation are altered coding sequences or expression by hundreds of genes to confer disease susceptibility. Several of these genes are associated either with etiology or with clinical response to treatment. Analysis of genomic profiles for the presence of drug targets and biomarkers should upgrade diagnostic accuracy, prevention measures, and target therapy. We discuss pharmacogenetics and pharmacogenenomics of oral anitdiabetic drugs. Studies that involve gene- -focused and larger-scale genomewide analyses, respectively, can provide specific new information on genetic variation affecting efficacy and individual susceptibility to side effects.

## 2. Genetic profiling, results from our study population in Taiwan

Up to 2011, at least 36 genes showing sequence variations associated with Type 2 diabetes across multiple populations emerged [6]. Candidate-gene studies lend strong evidence that common variants in genes have strong biological links to diabetes: genes of the peroxisome proliferator-activated receptor-r (PPARG) [7], potassium inwardly-rectifying channel J11 (KCNJ11) [8], transcription factor 2 isoform b (TCF2)[9], and Wolfram syndrome 1 (WFS1) [10]. Many additional genes have been linked to Type 2 diabetes in smaller-scale studies of single populations. GWAS have accelerated identification of T2D susceptibility genes, expanding the list from three in 2006 to over twenty in 2009 [11]. So far, the contribution to disease risk by any one of these factors is small (typically <1.5 fold increased risk). Despite these associations, data currently available lend inadequate support to management decisions for common forms of T2D. Also, though a great number of studies in various populations has suggested association between SNPs and T2D, findings from previous studies cannot be extrapolated to populations with different lifestyles, environment and SNP frequency.

Our research group attempted to discover susceptibility genes of T2D in Chinese population of Taiwan. This project recruited thousands of T2D patients aged 20 years and over, plus their comprehensive phenotype information, demorgraphic data, ethnicity, physical activity, medical history, health examination, blood and urine laboratory test, all collected upon enrollment. DNA was extracted and lymphoblastoid cell lines established for each patient. In 2010, the first GWAS based on our study population comprising 2,798 patients with T2D and 2,367 healthy controls was successfully published on *PLoS Genetics* [11]. We identified novel genetic susceptibility loci: *PTPRD* (protein tyrosine phosphatase receptor type D) and *SRR* (serine racemase), both associated with T2D in the Han Chinese population. Also, we confirmed involvement of *KCNQ1* , previously reported as associated with T2D in Japanese and European populations. Moreover, we conducted a replication study to confirm possible association studies between SNP susceptibility and T2D among Taiwanese. We searched literature to find SNPs related to T2D (*p* value less than 10^-5^) in Western and Asian countries with study design of GWAS or meta-analysis study. Several genes were replicated in our study population: *WFS1, CAMK1D, TSPAN8* and *HNF1B* , all related to pancreatic beta-cell development and function; *CDKAL1, IDE, WWOX* and *SRR* that relate to insulin production or secretion and more. According to our results from prior GWAS and replication study, we tried to build the prediction model with gene effect for T2D via logistic regression. In preliminary results, with the top 2,321 SNPs (from 20 genes) where *p* value≦10^-3^, area under curve (AUC) value reached 0.5254 in our study population. Result align with earlier studies proving genetic risk models with lower AUC values (0.55 e 0.68) than clinical models (AUC, 0.61 e 0.92) [12]. Incorporating genetic factors into clinical risk models only marginally improved and at times did not improve AUC value [13].

We showed effect of SNPs on diabetic retinopathy (DR) in Taiwanese population through GWAS or association studies. We identified 9 SNPs association for susceptibility to DR in five novel chromosomal regions and *ARHGAP22* (Rho GTPase-activating protein 22) and *PLXDC2* (plexin domain-containing 2). The last two are implicated in endothelial cell angiogenesis and increased capillary permeability [14]. Also, *PLEKHO2, PLEKHH1* [15] related to cell adhesion; *JPH* to calcium flux [16]; *TMEM217, MRPL14* and *GRIK2* [17] to glucocorticosteroid or amino acid metabolism were identified. In addition, we conducted replication studies to identify *VEGF, CHN2* [18], *MTHFR, EDIL3, CAMK4, CNTN5, HS6ST3* and *FMN1* . Predictive models or biomarkers related to DR were gleaned from literature. From such information, many traditional risk factors like age, gender, DM duration, fasting plasma glucose, glycosylated hemoglobin (HbA1c) and systolic blood pressure (SBP) have served to predict progression and severity of DR. Multifocal electroretinogram (mfERG IT Z-score) and blood biomarkers, such as lipid markers (HDL, LDL, Cholesterol and TG), apolipoprotein and cytokines were used in some studies. Among literature we searched, Fu et al.[19] and Nguyen et al. [20] evaluated genetic effect in their models, but their articles mentioned no difference between models with or without gene effect. According to information from literature, we chose clinical information, biological indicators and the SNPs associated with DR for logistic regression analysis. With 25 SNPs, from GWAS and replication with *p* value≦10^-2^, AUC value can rise from 0.7560 (from model including HbA1c and duration as variables only) to 0.8269 in our study population.

## 3. Pharmacogenomic studies

Individual traits that affect response to specific drugs have a pivotal role in personalized diabetes management. Rapid advances in knowledge of patient-specific pharmacology occur via the application of new molecular technology in gene-focused (pharmacogenetics) and hypothesis free approach, genomewide (pharmacogenomics) analyses. Both approaches examine variations in individual genetic makeup affecting efficacy and safety profiles of drugs. Since long-term hyperglycemia is a prominent contributor of micro- and macro-vascular complications, blood glucose control is a priority in T2D treatment. American Diabetes Association (ADA) Standards of Medical Care in Diabetes recommend lowering HbA1c to <7.0% in most cases to lower incidence of microvascular disease. For those with short disease duration, long life expectancy or no significant CVD, stringent HbA1c targets might be considered. Available options for glycemic management in T2D include not only exogenous insulin, but also a spectrum of pharmacologic agents whose actions include augmentation of insulin sensitivity, stimulation of insulin secretion, and slowing of intestinal glucose absorption. While glycemic control has improved over the past decade, about 40% of patients do not reach the desired HbA1c target of < 7%. Pharmacogenetic studies have been probed three classes of drugs commonly used in treatment of diabetes: i.e., metformin, sulfonylureas and thiazolidinediones (TZD). Numerous genetic markers have been identified so far [21,22], most for hypoglycemic agents in European populations, while pharmacogenomic advances in Chinese populations are quite limited. One should consider differences among studies—e.g., duration of treatment, mean age, ethnic/racial composition of cohort, T2D risk (gestational diabetes vs. impaired glucose tolerance)—as explanations for divergent association results.

## 4. Metformin

This biguanide, an insulin sensitizer, decreases glucose production in the liver, enhancing insulin sensitivity and peripheral glucose uptake. It is the most widely used first-line T2D drug, but only 60-65% of patients attain desired glycemic control or HbA1c goal of less than 7%. Metformin suppresses hepatic gluconeogenesis by activating AMP-activated protein kinase (AMPK) that inhibits expression of hepatic gluconeogenic genes *PEPCK* and Glc-6-pase by raising expression of small heterodimer partner (SHP). It is not metabolized but excreted unchanged in urine by active tubular secretion [23]. Various proteins relate to oral absorption, hepatic uptake and renal elimination [24]. Plasma membrane monoamine transporter (PMAT, encoded by *SLC29A4* (solute carrier family 29 member 4) gene), expressed on the luminal side of enterocytes [25], relates to intestinal absorption of metformin. Organic cation transporter 1 (OCT1, encoded by *SLC22A1* (solute carrier family 22 member 1) gene) is necessary for metformin transport into the liver and subsequent metformin activity. Organic cation transporter 2 (OCT2 , encoded by *SLC22A2* (solute carrier family 22 member 2)) is expressed chiefly at basolateral membrane of the renal epithelium and transport of metformin into proximal tubule cells [26]. Multidrug and toxin extrusion transporter 1 (MATE1, encoded by *SLC47A1* (solute carrier family 47, member1)) and MATE2-K (encoded by *SLC47A2* (solute carrier family 22 member 2)), located in the apical membrane of renal proximal tubule cells, facilitate metformin excretion from tubular cells [27].

Pharmocogenetic researchers link polymorphisms in genes *SLC22A1*[28], *SLC22A2*[29], *SLC47A1*[30], *SLC47A2*[31], with altered metformin response. Polymorphisms of candidate genes encoding OCT3 (*SLC22A3* ) [32] and PMAT (*SLC29A4* ) [25] transporters were related to modulate metformin pharmacokinetics and response. Recently, the first GWAS on glycemic response to metformin in 1,024 Scottish individuals with T2D was performed and replicated in two cohorts: 1,783 Scottish individuals and 1,113 individuals from the UK Prospective Diabetes Study [33]. The study identified a common rs11212617 A>C SNP at a locus containing *ATM*, ataxia telangiectasia mutated gene associated with treatment success. The researchers also demonstrated ATM inhibition with KU-55933 attenuating phosphorylation and AMP-activated protein kinase in response to metformin in a rat hepatoma cell line.

## 5. Sulfonylureas

This oldest oral agent class is thought to stimulate insulin secretion by closing ATP-sensitive potassium channels in pancreatic beta-cells. The blood glucose-lowering drug belongs to insulin secretagogues expediting production and secretion of insulin. Common sulfonylureas tolutamide, gliclazide, glibenclamide and glimepiride effectively control glucose levels but with modest weight gain and risk of hypoglycaemia. Most patients respond well, 10-20% of persons treated do not attain adequate glycemic control, 5-10% initially responding to sulfonylurea subsequently lose ability to maintain near-normal glycemic level. This implies genetic factors linked with treatment efficacy. Sulfonylureas release insulin from pancreatic beta cell by first binding to high-affinity plasma membrane receptor (SUR1) coupled with an ATP-dependent K channel (KAPT). Polymorphisms in drug target genes (ATP-binding cassette transporter subfamily C member 8 (*ABCC8* ) and potassium inwardly-rectifying channel, subfamily J, member 11 (*KCNJ11* ) have recently shown linkage with variability in response to sulfonylurea drugs in T2D cases [34]. *KCNJ11* gene, located on the short arm of chromosome 11, encodes pore-forming subunit of the ATP-sensitive potassium channel Kir6.2 in pancreatic β-cells. Gain-of-function mutations in *KCNJ11* open the potassium channel and inhibit depolarization of β-cells, leading to a defect in insulin secretion. Most oral hypoglycemic agents are metabolized in the liver by cytochrome P450 (CYP) enzymes; effect on response to treatment with sulfonylurea was probed. Variations in *CYP2C9* link with impaired metabolism and reduced oral clearance of sulfonylureas. Patients carrying CYP2C9*2 and *3 required lower doses and were more likely to achieve glycemic goals (including HbA 1c), but they showed higher risk of mild or severe hypoglycemia, major adverse effect of these drugs. Also, polymorphisms of gene in hepatocyte nuclear factor-1α (*HNF-1α*), a transcription factor vital to beta cell development and function, and *TCF7L2* that encodes transcription factor (Tcf-4), involved in regulation of cellular proliferation and differentiation, were investigated. Pearson et al. [35] found patients with *HNF-1α* gene mutations supersensitive to treatment with sulfonylureas but responded poorly to treatment with metformin. In addition, research has shown that individuals with the *TCF7L2* risk genotype respond poorly to sulfonylureas [36].

Meglitinides, a class of insulin secretagogues, stimulate insulin release via similar mechanisms as Sulfonylureas but may associate with less hypoglycaemia. Yet they require more frequent dosing. Common meglitinides are nateglinide, repaglinide and mitiglinide. Polymorphisms linked with variable meglitinide response include *CYP2C8* that associates with efficacy of repaglinide, rosiglitazone and pioglitazone, as well as *CYP2C9* affecting nateglinide efficacy. As for drug distribution, polymorphisms in genes encoding drug transporters play key roles in efficacy. Variants of *SLCO1B1* that encodes organic anion transporting polypeptide 1B1 (OATP1B1), impacted efficacy of repaglinide, nateglinide, rosiglitazone and pioglitazone. Researchers pay great heed to pharmacodynamics of repaglinide in Chinese patients recently. Numerous markers link with repaglinide effect: e.g., genes encoding P-glycoprotein transporter (*MDR1* , 2677T/A), voltage gated K+channel (*KCNQ1* , rs2237892 (C>T) and rs2237895 (C>A)), zinc transporter solute carrier family 30 member 8 (*SLC30A8* , rs13266634 (C > T) and rs16889462 (G>A)), *KCNJ11* (Lys23Glu), *TCF7L2* (rs290487 (C/T) and nicotinamide phosphoribosyltransferase (*NAMPT* , -3186C>T).

## 6. Thiazolidinediones (TZDs)

These peroxisome proliferators activate receptor γ (PPAR-γ) to improve insulin sensitivity in skeletal muscles and reduce hepatic glucose production. PPARG gene encodes peroxisome proliferator-activated receptor γ, a Type II nuclear receptor that plays a fundamental role in adipogenesis and insulin sensitivity by regulating transcriptional activity of various genes. TZDs sharply reduce triglyceride content in adipose tissue, skeletal muscles and liver while raising leptin concentration [21]. Together, these changes decrease circulating free fatty acids (FFA), which reduces FFA-induced insulin resistance in skeletal muscles. Among TZDs, rosiglitazone, pioglitazone and troglitazone demonstrably improve glycemic control and may slow progression of β-cell failure. Most studies focus on variants of PPAR-γ and its harboring genes in TZDs therapy outcomes. Polymorphisms of *PPARG, PGC-1alpha, adiponectin , leptin, TNF alpha* and *CYP2C8* relate to rosiglitazone therapy. Yet due to serious side effects of rosiglitazone, including risk of myocardial infarction and death from cardiovascular, the only TZD still available on the market is pioglitazone. Studies link polymorphism of resistin (SNP-420) with reduction of FPG and HOMA-IR (Homeostasis Model of Assessment-Insulin Resistance) by pioglitazone [37]. Response to pioglitazone treatment on glycemic control (reduction fasting blood glucose > 10% after 10 weeks treatment) is related to the polymorphism of gene encoding lipoprotein lipase (LPL, Ser447X), enzyme responsible for processing of triglyceride-rich lipoproteins [38]. For TZDs treatment, a side effect is high rate of fluid retention and peripheral edema. Chang et al. [39] proved female gender, older age and genetic polymorphism as contributing factors. The *AQP2*polymorphism (rs296766) coding aquaporin-2 (vasopressin-regulated water channel), and polymorphism of *SLC12A1* (solute carrier protein family 12 group A, member one) gene (rs12904216) coding the sodium-potassium-2 chloride transporter (*NKCC2*) with a key role in electrolyte movement across epithelia, are reported as related to TZDs-associated edema.

## 7. Conclusion

It is still far from routine clinical practice to use genotypic markers for type 2 diabetes. But it could be anticipated that learning about these and additional genetic determinants of the risks, coupled with proteomic and metabolomic analyses, will show potential to direct individualized decisions in deciding relative efficacy and dosage profiles of oral antidiabetic drugs in prevention and management of T2D and diabetic complications. Still, results from large cohort studies must provide fundamental data that can be used to profile risk factors and discover novel therapeutic targets. Likewise, pharmacoeconomics will be considered in personalized medicine.

**Table.** Gene polymorphisms involved in pharmacogenetics of oral anti-diabetes drugs

	Drug	Gene	Variation
Insulin Sensitizers			
Biguanides	Metformin	SLC22A1	rs12208357, rs34130495, rs72552763, rs34059508 [40]
			rs12208357, rs72552763 [28]
			rs622342 [41]
		SLC22A2	rs201919874, rs145450955, rs316019 [42]
			rs316019[43]
		SLC47A1	rs2289669 [44], rs8065082 [30]
		SLC47A2	rs12943590 [31]
		ATM	rs11212617 [33]
TZD	Pioglitazone	PPARG	rs1801282 [45]
	Rosiglitazone	PGC-1α	rs2970847, rs8192678[46]
	Troglitazone	Resistin	rs1862513 [37]
		Leptin	rs7799039 [47]
		TNF-α	rs1800629 [47]
		CYP2C8	rs10509681 [48]
Insulin Secretagogues			
K+ ATP	Sulphonylurea	KCNJ11	rs5219 [49]; rs5210 [50]
		ABCC8	rs757110[50]
			rs1799854, rs1799859 [51]
		KCNQ1	rs163184 [52]
		TCF7L2	rs7903146 [53]
			rs12255372, rs7903146 [36]
		CYP2C9	rs1057910 [54]
			rs1799853, rs1057910 [55]

## Acknowledgments

This study is supported in part by China Medical University (CMU102-N-01).
